# Monocarboxylate transporter 4 (MCT4) and CD147 overexpression is associated with poor prognosis in prostate cancer

**DOI:** 10.1186/1471-2407-11-312

**Published:** 2011-07-25

**Authors:** Nelma Pértega-Gomes, José R Vizcaíno, Vera Miranda-Gonçalves, Céline Pinheiro, Joana Silva, Helena Pereira, Pedro Monteiro, Rui M Henrique, Rui M Reis, Carlos Lopes, Fátima Baltazar

**Affiliations:** 1 Life and Health Sciences Research Institute (ICVS), School of Health Sciences, University of Minho, Braga, Portugal; 2 ICVS/3B's - PT Government Associate Laboratory, Braga/Guimarães, Portugal; 3 Department of Pathology, Centro Hospitalar do Porto, Portugal; 4 CBMA - Centro de Biologia Molecular e Ambiental, Universidade do Minho, Campus de Gualtar 4710-057 Braga; 5 Department of Pathology, Centro Hospitalar do Alto Ave, Guimarães, Portugal; 6 Department of Genetics and Pathology, Portuguese Oncology Institute-Porto, Porto, Portugal; 7 Molecular Oncology Research Center, Barretos Cancer Hospital, Barretos, São Paulo, Brazil

## Abstract

**Background:**

Monocarboxylate transporters (MCTs) are transmembrane proteins involved in the transport of monocarboxylates across the plasma membrane, which appear to play an important role in solid tumours, however the role of MCTs in prostate cancer is largely unknown. The aim of the present work was to evaluate the clinico-pathological value of monocarboxylate transporters (MCTs) expression, namely MCT1, MCT2 and MCT4, together with CD147 and gp70 as MCT1/4 and MCT2 chaperones, respectively, in prostate carcinoma.

**Methods:**

Prostate tissues were obtained from 171 patients, who performed radical prostatectomy and 14 patients who performed cystoprostatectomy. Samples and clinico-pathological data were retrieved and organized into tissue microarray (TMAs) blocks. Protein expression was evaluated by immunohistochemistry in neoplastic (n = 171), adjacent non-neoplastic tissues (n = 135), PIN lesions (n = 40) and normal prostatic tissue (n = 14). Protein expression was correlated with patients' clinicopathologic characteristics.

**Results:**

In the present study, a significant increase of MCT2 and MCT4 expression in the cytoplasm of tumour cells and a significant decrease in both MCT1 and CD147 expression in prostate tumour cells was observed when compared to normal tissue. All MCT isoforms and CD147 were expressed in PIN lesions. Importantly, for MCT2 and MCT4 the expression levels in PIN lesions were between normal and tumour tissue, which might indicate a role for these MCTs in the malignant transformation. Associations were found between MCT1, MCT4 and CD147 expressions and poor prognosis markers; importantly MCT4 and CD147 overexpression correlated with higher PSA levels, Gleason score and pT stage, as well as with perineural invasion and biochemical recurrence.

**Conclusions:**

Our data provides novel evidence for the involvement of MCTs in prostate cancer. According to our results, we consider that MCT2 should be further explored as tumour marker and both MCT4 and CD147 as markers of poor prognosis in prostate cancer.

## Background

Increased glucose consumption is a hallmark of malignant cells, which is responsible for energy production from glycolysis [1]. Most malignancies rely on this pathway for rapid proliferation even in the presence of oxygen, leading to production of large amounts of acids, mainly lactic acid [1,2]. Consequently, the high glycolytic phenotype induces an acidic tumour environment, which is associated with the increase of several malignant features including cellular migration, invasion and metastisation [2].

In order to prevent cell death by cellular acidosis, tumour cells increase proton efflux through pH regulators such as proton-pumps, sodium-proton exchangers, bicarbonate transporters and monocarboxylate transporters (MCTs) [3]. MCTs are proteins that facilitate the transmembrane transport of short-chain fatty acids, such as pyruvate and lactate, coupled with a proton. In glycolytic tumours, they promote the efflux of lactic acid, constituting important players in the maintenance of tumour intracellular pH, as well as in the maintenance of the high rates of glycolysis [4,5]. Therefore, MCTs play a central role in tumour metabolism and, as a result, constitute attractive targets in cancer therapy which have not been explored yet.

We and others have shown evidence for the upregulation of MCTs in several solid tumours, such as colorectal carcinomas [6], uterine cervix carcinomas [7], melanomas [8], breast carcinomas [9,10] and lung tumours [11]. However, in prostate carcinoma, the role of MCTs is largely unknown. To the best of our knowledge, we were the first to report MCT expression in prostate cancer [12,13], however, there is a recent study [14] evaluating the role of MCTs in prostate drug resistance and progression but this study does not evaluate neither MCT isoform 2 (MCT2) nor gp70, its known chaperone.

MCT expression appears to be influenced by altered physiologic conditions, however the underlying molecular events involved in MCT regulation are still poorly understood. Recently, it was demonstrated that proper expression and activity of MCT1 and MCT4 requires co-expression of CD147, also known as EMMPRIN or basigin [15-17]. On the other hand, *in vitro *studies showed that maturation and cell surface expression of CD147 is also dependent on MCT1 and MCT4 expressions [18,19]. MCT2 expression and activity depends on a different chaperone known as gp70.

CD147 alone has already been described as a key element in oncogenesis by stimulating the synthesis of several matrix metalloproteinases, leading to enhanced tumour cell invasion [20,21]. This protein is described to be up-regulated in tumours, including prostate cancer, where it has been identified as an unfavourable prognosis marker [22-25]. However, the role of CD147/MCT co-expression in prostate cancer is far from understood.

The aim of the present study was to assess the role of MCTs in prostate cancer, by comparing the immunohistochemical expression of the MCT isoforms 1, 2 and 4, along with CD147 and gp70, in normal prostatic tissue, adjacent non-neoplastic tissue, PIN lesions and neoplastic tissues in a large series of prostate samples organized into tissue microarrays (TMAs), and evaluating their clinico-pathological value.

## Methods

### Case selection and TMA construction

Prostate tissues were obtained from 171 patients with a median age of 64 years old (range 46-74), who performed radical prostatectomy between 1993 and 2003. Samples and clinico-pathological data were retrieved from the files of the Department of Pathology, Centro Hospitalar do Porto and Centro Hospitalar do Alto Ave-Guimarães, and organized into 13 tissue microarray blocks (TMAs).

Prior to TMA construction, hematoxylin and eosin (H&E) tumour sections of each specimen of radical prostatectomy were re-assessed using the 2005 modified Gleason and 2010 p TNM AJCC classification [26,27]. Representative areas of adjacent non-neoplastic prostate tissue, PIN lesions and prostate cancer were selected. Adjacent non-neoplastic tissue samples and PIN lesions were selected from the peripheral zone of prostate were cancer develops. Each case was represented in the TMA by three cores (1 mm diameter) with 0.8 mm from core centre to core centre, and precisely deposited into a recipient paraffin block, using a TMA workstation (TMA builder, Beecher Instruments Inc. Technology). 4 μm tissue sections were used for immunohistochemistry (IHC) and H&E-stained section from each TMA block was reviewed to confirm the presence of morphological representative areas of the original tissues.

Normal prostate tissue was obtained from cystoprostatectomy cases and immunohistochemical expression was performed in the entire section of the fragments.

### MCT and CD147 immunohistochemistry

IHC for MCTs was performed according to avidin-biotin-peroxidase complex principle (R.T.U. Vectastain Elite ABC Kit [Universal], Vector Laboratories, Burlingame, CA), with the primary antibodies for MCT1 (AB3538P, Chemicon International, Temecula, CA), MCT2 (sc-14926, Santa Cruz Biotechnology, Santa Cruz, CA) and MCT4 (AB3316P, Chemicon International, Temecula, CA), diluted 1:200, as previously described [6,7,9,10].

CD147 and gp70 IHC was performed according to the same principle (Ultravision Detection System Antipolyvalent, horseradish peroxidase; Lab Vision Corporation), with the primary antibody diluted 1:750, as previously described [9,10] for CD147 (18-7344, ZYMED Laboratories Inc., South San Francisco, CA) and diluted 1:100 for gp70 (HPA017740, Atlas Antibodies).

Negative controls were performed with appropriate serum controls for the primary antibodies (X0907 and N1699, Dako, Carpinteria, CA). Normal colon, kidney and skeletal muscle tissue were used as positive controls for MCT1, MCT2 and MCT4, respectively, cervical squamous carcinoma for CD147 and seminal vesicle for gp70. Tissue sections were counterstained with hematoxylin.

### Immunohistochemical evaluation

Immunoreaction in TMA sections was evaluated for cytoplasmic and/or plasma membrane staining. Shortly, sections were scored semi-quantitatively as follows: 0: 0% of immunoreactive cells; 1: < 5% of immunoreactive cells; 2: 5-50% of immunoreactive cells and 3: > 50% of immunoreactive cells. Also, intensity of staining was scored semi-quantitatively as follows: 0: negative; 1: weak; 2: moderate and 3: strong. The final score was defined as the sum of both parameters (extension and intensity), and grouped as negative (scores 0-3) and positive (scores 4-6). Discordant results in different cores of the same case were scored as follows: average of extension plus highest intensity score. Immunohistochemical evaluation was performed by two independent and experienced pathologists (JRV, PM), who were blind to the clinico-pathological data of the patients. Discordant results were discussed in a double-head microscope. Since staining was different among the positive cases, to further clarify the significance of the immunoexpression of MCTs and CD147 in prostate carcinoma, we categorized the cases in two groups: intermediate score group (ISG, score 4) and high score group (HSG, scores 5-6).

### Statistics

Statistical analysis was performed using the SPSS statistical software (version 17.0, SPSS Inc., Chicago, IL, USA). All comparisons were examined for statistical significance using Pearson's chisquare *(χ*^2^) test, being the threshold for significance *p *< 0.05.

### Ethics

The work has been approved by DEFI (Departamento de Ensino Formação e Investigação) Ethics Committee of Centro Hospitalar do Porto ref. no. 017/08(010-DEFI/015-CES).

## Results

### MCT, CD147 and gp70 expressions in prostate tissues

**Figure 1:**
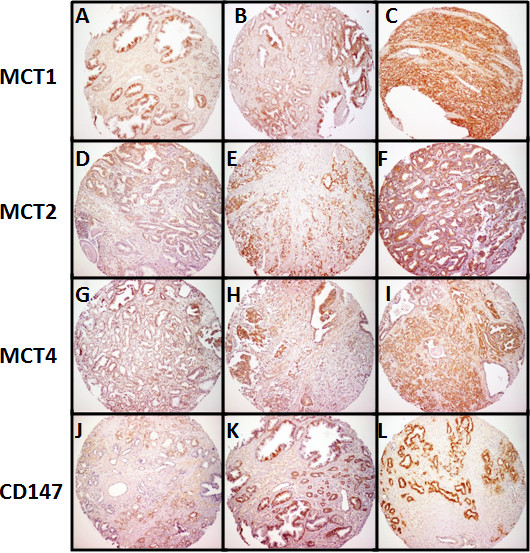
**Expression of MCT1, MCT2, MCT4 and CD147 in prostate cancer tissue microarrays**. Representative images of intensity score 1 (weak) for MCT1 (A), MCT2 (D), MCT4 (G) and CD147 (J), intensity score 2 (moderate) for MCT1 (B), MCT2 (E), MCT4 (H) and CD147 (K) and intensity score 3 (strong) for MCT1 (C), MCT2 (F), MCT4 (I) and CD147 (L) immunostaining for positive cases of prostate carcinoma (score ≥ 4).

A total of 346 prostate samples organised into TMAs (tissue microarrays), including 135 non-neoplastic, 40 PIN lesions and 171 neoplastic tissues were analysed for MCT1, MCT2, MCT4, CD147 and gp70 expressions. Also, 14 normal prostate tissues were analysed for MCTs, CD147 and gp70 expressions. We used a combined scoring system, previously described [6,7,9,10]. To better illustrate the scoring system used, representative images of positive cases with intensity score 1 (weak), score 2 (moderate) and score 3 (strong), for MCT1, MCT2, MCT4 and CD147 staining are shown in Figure 1.

**Figure 2:**
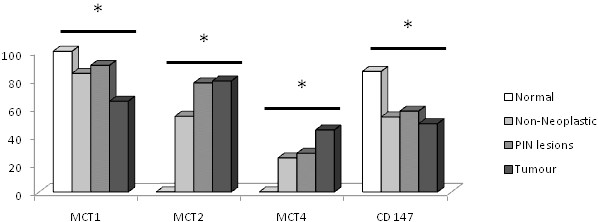
**Frequency of MCTs and CD147 expressions in normal prostate, non-neoplastic, PIN lesions and tumour samples**. In general, there is an increase in both MCT2 and MCT4 expressions from non-neoplastic (normal or adjacent) to tumour tissues, while a decrease is observed for MCT1 and CD147 expression in the transition from non-neoplastic (normal or adjacent) to prostate tumour tissue. See text for detail. * *p *< 0.05 (non-neoplastic adjacent, PIN and tumour tissue compared to normal tissue).

Figure 2 summarises MCT and CD147 expressions in normal, adjacent non-neoplastic, PIN lesions and tumour tissues. A significant increase in both MCT2 and MCT4 expressions was observed from non-neoplastic (normal or adjacent) to tumour tissues (*p *< 0.001, for both) while a decrease was observed for MCT1 expression in the transition from normal or adjacent non-neoplastic to prostate tumour tissue (*p *= 0.003 and *p *< 0.001, respectively). CD147 expression decreased from normal to tumour tissue (*p *= 0.006), however, no significant differences were observed when compared to adjacent non-neoplastic tissue (*p *= 0.236). For MCT1 expression, we observed a decrease from PIN lesions to malignant glands (*p *< 0.001) with no significant differences between normal or adjacent non-neoplastic tissue and PIN lesions (*p *= 0.545 and *p *= 0.063, respectively). For MCT2, there was an increase from both normal and adjacent non-neoplastic tissue to PIN lesions (*p *< 0.001 and *p *= 0.005, respectively) whereas no significant differences were observed between PIN lesions and tumour (*p *= 0.605). There was a significant increase in MCT4 expression from normal to PIN lesions (*p *= 0.024) and from PIN lesions to tumour (*p *= 0.022) but not between adjacent non-neoplastic tissue and PIN lesions (*p *= 0.410). For CD147, there was a significant decrease from normal tissue to PIN lesions (*p *= 0.043) but no difference between adjacent non-neoplastic tissue and PIN lesions (*p *= 0.389). No differences were observed between CD147 expression in PIN lesions and tumour (*p *= 0.180). Gp70 was negative in all normal, adjacent non-neoplastic and PIN lesions and only a very small percentage of cases (n = 4) were positive in tumours (data not shown).

**Figure 3:**
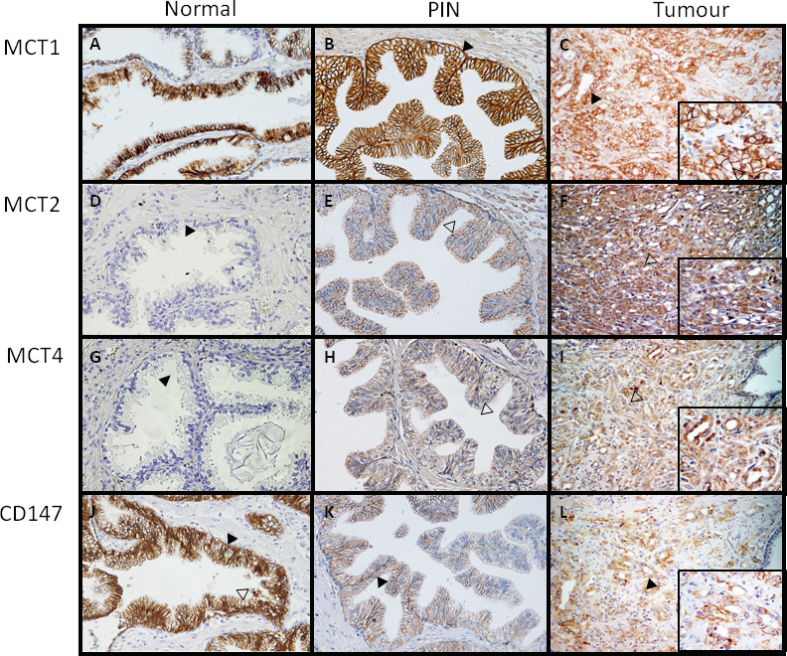
**Immunohistochemical expression of MCTs and CD147 in normal, PIN lesions and neoplastic prostate tissue**. MCT1 immunoreactivity in normal glands (A), PIN lesions (B) and malignant glands (C) was observed in the basal and lateral epithelial cell membranes (solid arrow head) with no immunoreaction in the apical zone (open arrow head). MCT2 immunoreactivity in normal (D), PIN lesions (E), malignant glands (F) was mainly observed in the cytoplasm of epithelial cells, being absent in the normal glands (solid arrow head) but with strong granular pattern in malignant glands and PIN lesion (open arrow head). MCT4 immunoreactivity in normal glands (G), PIN lesions (H) malignant glands (I) was observed in the cytoplasm, with granular appearance (open arrow head), being absent in normal glands (solid arrow head). CD147 immunoreactivity in normal glands (J), PIN lesions (K) and malignant glands (L) was observed in the basal and lateral epithelial cell membranes (solid arrow head), with no immunoreaction in the apical zone (open arrow head). Main pictures are at 200× magnification and insets are at 400×.

Figure 3 shows representative immunohistochemical reactions for MCT1, MCT2, MCT4 and CD147 in normal, PIN lesions and tumour tissue. Staining for MCT1 and CD147 was mainly observed in the basal and lateral epithelial cell membranes, with negative immunoreactions in the apical zone of both normal glands (Figure 3A, J) PIN lesions (Figure 3B, K) and neoplastic cells (Figure 3C, L). MCT2 and MCT4 staining was cytoplasmic, with granular appearance, which was more evident for MCT2 (Figure 1F). Due to the epithelial nature of prostate carcinoma, positive immunoreactions were only considered for staining in epithelial cells.

As stated in Materials and Methods section, we stratified the positive cases into two groups, ISG (intermediate score group) and HSG (high score group). Although the number of normal prostate tissue cases is small, the differences between normal and tumour cases was evident, however, this difference was not so clear between adjacent non-neoplastic tissue and tumour. Thus, we compared the expression of the proteins in the ISG and HSG (Table 1). For both MCT1 and MCT4, there was a significant difference between neoplastic and adjacent non-neoplastic tissues only for the HSG. For MCT2, HSG predominated in neoplastic cells, whereas ISG was more frequent in adjacent non-neoplastic tissue. There were no differences in the distribution of CD147 final score between neoplastic and adjacent non neoplastic tissues.

**Table 1:** Distribution of positive final immunohistochemical score of monocarboxylate transporters (MCTs) and CD147 in adjacent non-tumoural (NT) and tumour tissue (T) of prostate samples

			ISG (%)		HSG (%)	
		n	(Score 4)	*p*	(Scores 5-6)	*p*
**MCT1**				0.500		**0.002**
	**NT**	**121**	17.4		66.9	
	**T**	**166**	18.0		46.4	
**MCT2**				**0.048**		**< 0.001**
	**NT**	**132**	37.9		16.7	
	**T**	**166**	26.0		53.0	
**MCT4**				0.474		**< 0.001**
	**NT**	**128**	16.4		7.8	
	**T**	**168**	20.8		23.2	
**CD147**				0.211		0.153
	**NT**	**134**	11.9		41.1	
	**T**	**167**	16.7		32.9	

**ISG**, intermediate score group; **HSG**, high score group.

We further assessed the association between CD147 and MCT expressions in the prostate cancer tissues (Table 2). This analysis showed that CD147 correlated with both MCT1 and MCT4 (*p *< 0.001 for both), but not with MCT2. Figure 3 (C, I and L) shows staining for MCT1, MCT4 and CD147 in the same tumour area of one prostate tumour case, in which positive cells for the three proteins can be seen. No associations between gp70 and MCTs were found (data not shown).

**Table 2:** Association between MCT1, MCT2, MCT4 and CD147 expressions in prostate tumours

	*MCT1 *			*MCT4*			*MCT2*		
	
	n	Positive (%)	*p*	n	Positive (%)	*p*	n	Positive (%)	*p*
**CD147**			**< 0.001**			**< 0.001**			0.184
Negative	**59**	20.3		**84**	26.7		**35**	40.0	
Positive	**107**	64.5		**83**	63.0		**131**	50.4	

### Associations between MCTs and CD147 expressions and the clinic-pathological data

Assessment of association between MCTs and CD147 expressions and the clinico-pathological data is presented in Table 3. We found positive associations between MCT1 expression in the HSG and higher PSA levels (*p *= 0.016), absence of perineural invasion (*p *= 0.036) and presence of biochemical recurrence (*p *= 0.047). For MCT2, there was only an association with lower age at diagnosis for ISG (*p *= 0.023). MCT4 expression in the HSG was associated with higher age (*p *< 0.001), higher PSA levels (*p *< 0.001), advanced tumour stage (pT3, *p *< 0.001), higher Gleason score (*p *= 0.011), presence of perineural invasion (*p *= 0.011) and presence of biochemical recurrence (*p *< 0.001). CD147 expression in the HSG correlated with higher age (*p *< 0.001), higher PSA levels (*p*<0.001), advanced tumour stage (pT3, *p *< 0.001), higher Gleason score (*p *= 0.012), presence of perineural invasion (*p *= 0.021) and presence of biochemical recurrence (*p *< 0.001). No associations between gp70 and clinico-pathological data were found (data not shown).

**Table 3:** Correlations between monocarboxylate transporters (MCTs) and CD147 expressions in prostate tumour samples and clinico-pathological data

			MCT1				MCT2			MCT4					CD147		
	
Variable	n	ISG %(n)	*p*	HSG %(n)	*p*	ISG %(n)	*p*	HSG %(n)	*p*	ISG %(n)	*p*	HSG %(n)	*p*	ISG %(n)	*p*	HSG %(n)	*p*
																	
**Age**			0.391		0.073		**0.023**		0.117		0.247		**< 0.001**		0.518		**< 0.001**
> 64	**93**	19.4 (18)		40.9 (38)		32.6 (30)		48.4 (45)		18.5 (17)		8.6 (8)		17.2 (16)		16.1 (15)	
> 64	**73**	16.4 (12)		53.4 (39)		17.8 (13)		58.9 (43)		24.0 (18)		42.3 (31)		16.2 (12)		54.1 (40)	
																	
**PSA (ng/ml)**			0.414		**0.016**		0.085		0.250		0.060		**< 0.001**		0.529		**< 0.001**
> 11	**116**	19 (22)		40.5 (47)		29.6 (34)		50.9 (116)		17.2 (20)		11.1 (13)		17.1 (20)		16.2 (19)	
> 11	**50**	16 (8)		60.0 (30)		18.0 (9)		58.0 (50)		29.4 (15)		51.0 (26)		16.0 (8)		72.0 (36)	
																	
**pT**			0.511		0.145		0.528		0.297		0.210		**< 0.001**		0.396		**< 0.001**
2	**130**	18.5 (24)		43.8 (57)		26.4 (34)		51.5 (67)		19.2 (25)		14.5 (19)		16.0 (21)		24.4 (32)	
3	**36**	16.7 (6)		55.6 (20)		25.0 (3)		58.3 (21)		27.0 (10)		54.1 (20)		19.4 (7)		63.9 (23)	
																	
**Gleason score**			0.251		0.170		0.386		0.857		0.692		**0.011**		0.850		**0.012**
< 7	**57**	15.8 (9)		52.6 (30)		31.6 (18)		54.4 (31)		24.6 (14)		10.5 (6)		15.8 (9)		21.1 (12)	
7	**100**	21.0 (21)		41.0 (41)		22.2 (22)		53.0 (53)		18.8 (19)		28.4 (29)		17.8 (18)		36.6 (36)	
> 7	**9**	0 (0)		66.7 (6)		33.3 (3)		44.4 (4)		22.2 (2)		44.4 (4)		11.1 (1)		66.7 (6)	
																	
**Perineural**																	
**Invasion**			0.525		**0.036**		0.503		0.259		0.397		**0.011**		0.531		**0.021**
Absent	**52**	17.3 (9)		57.7 (30)		26.9 (14)		57.7 (30)		23.1 (12)		11.5 (6)		17.3 (9)		21.2 (11)	
Present	**114**	18.4 (21)		41.2 (47)		25.7 (29)		50.9 (58)		20.0 (23)		28.4 (33)		16.5 (19)		38.3 (44)	
																	
**Biochemical**																	
**Recurrence**			0.434		**0.047**		0.597		0.089		0.200		**< 0.001**		0.489		**< 0.001**
Absent	**139**	18.7 (26)		43.2 (60)		26.1 (36)		50.4 (70)		19.4 (27)		17.1 (24)		16.4 (23)		26.4 (37)	
Present	**27**	14.8 (4)		63.0 (17)		25.9 (7)		66.7 (18)		28.6 (8)		53.6 (15)		18.5 (5)		66.7 (18)	

**ISG**, intermediate score group; **HSG**, high score group.

## Discussion

Prostate cancer remains a major concern in public health, being one of the most prevalent tumours and the second leading cause of cancer death in men [28]. Thus, it is important to elucidate its biology in order to find new markers and more efficient treatments.

Experimental evidence points at MCTs as potential targets for cancer therapy [29,30], however, the role of these membrane proteins in prostate cancer is poorly understood. Thus, the present work is an attempt to shed light into the involvement of MCTs in prostate cancer. With this purpose, we analysed the expressions of MCT1, MCT2, MCT4, CD147 and gp70 in a series of prostate samples, including normal, adjacent non-neoplastic, PIN lesions and neoplastic tissues.

### MCT1, MCT2, MCT4 and CD147 are differentially expressed in non-neoplastic, PIN lesions and neoplastic prostate tissues

In the present study, a general decrease in MCT1 and CD147 levels from normal prostate tissue to PIN lesions and prostate carcinoma was observed. Since their expression in normal prostate epithelium is high, it appears that they have an important role in normal tissue and are downregulated in prostate cancer cells, where other adaptive mechanisms may be activated. Importantly, there was an increase in both MCT2 and MCT4 expressions from normal to PIN lesions and tumour samples. In part, our results contradict the ones of Hao et al. [14], which stated that both MCT1 and MCT4 are upregulated in prostate cancer tissue, however the percentage of MCT4 positivity for pT3 tumours (around 40%) is similar to ours (44% tumour positive cases). Since the number of cases analysed by these authors is only slightly smaller than ours, the differences observed may be due to the different antibodies used. The specificity of our antibodies was tested by western-blot and more recently by RNAi for MCT1 (data not shown). We also observed frequent and clear MCT1 membrane expression, while these authors state that MCT1 staining was mostly cytoplasmic. The granular appearance of MCT2 and MCT4 expression in the cytoplasm of prostate tumour cells, as well as the predominance of the strong immunostaining scores suggests that these MCT isoforms could have important functions in some organelle membranes, possibly playing a role in the metabolism of prostate tumour cells.

Importantly, MCT2 was the only marker which allowed distinction between adjacent non-neoplastic tissue and PIN lesions. For MCT1, MCT4 and CD147, expression was similar in both. Despite apparently normal to the Pathologist, adjacent non-neoplastic glands already present alterations from normal tissue.

### Monocarboxylate transporters 1 and 4 expression is associated with CD147 in prostate cancer

A close association between CD147 expression and both MCT1 and 4 was found, similar to the findings of Hao et al. [14]. Additionally, we detected no association with MCT2, supporting CD147 as chaperone for both MCT1 and MCT4 but not with MCT2 in prostate cancer. Studies of CD147 in paraffin-embedded specimens of prostate cancer are not many, however they describe overexpression of CD147 in prostate cancer, reporting expressions between 60 to 80% [22-25]. In the present study, we found around 50% of prostate tumour cases expressing CD147 and this expression was significantly different from the normal prostate tissue. To the best of our knowledge, our study has the biggest sample size and the number of non-neoplastic samples analysed is similar to the tumour samples.

Gp70 was only expressed in very few cancer cases and there was no association with either MCT isoforms. This result was surprising, however, since MCT2 was not present in the plasma membrane, the mechanism of regulation might be different. In addition, as described for CD147 and MCT isoforms 1 and 4 [31], there might be other chaperones involved in MCT2 regulation.

### MCT4 and CD147 overexpression is associated with poor prognosis in prostate cancer

Assessment of associations between MCTs and CD147 expression and clinico-pathological data, revealed some important associations. In accordance to the work of other groups, we found positive associations between both MCT4 and CD147 expressions and higher PSA levels, advanced tumour stage and higher Gleason score [14,22-25]. However, regarding tumour stage, the difference in MCT4 expression in the report of Hao and collaborators [14], appears to be only from pT1 to pT2 and 3 since the levels of the last two appear to be similar. In our series we do not have pT1 stage cases, which makes the comparison more difficult. Additionally, we found associations between MCT1 expression and higher PSA levels, absence of perineural invasion and presence of biochemical recurrence, as well as between both MCT4 and CD147 and presence of perineural invasion and biochemical recurrence, which to the best of our knowledge, was not described by others. MCT2 also correlated with lower age at diagnosis, while both MCT4 and CD147 were associated with higher age at diagnosis, which significance is not known.

The differences observed between our study and others, besides the use of different antibodies, might be also due to the diverse immunohistochemical assessment, while in the present study we considered both intensity and percentage of immunoreactive cells, other authors considered only either intensity or extension of staining [14,22-25]. We believe that evaluating two parameters instead of one, decreases subjectivity and will have higher biological significance. Moreover, our non-neoplastic tissue was selected from the peripheral zone of the prostate in which prostate cancer is diagnosed and this may also have contributed to the differences observed.

Overall, we found no important clinicopathological associations with MCT2 but tumours that are highly positive (HSG) for MCT1, MCT4 or CD147, seem to exhibit a more aggressive behaviour, especially MCT4 and CD147 which correlated with higher PSA levels, higher pT stage, higher Gleason score, presence of perineural invasion and biochemical recurrence. However, to elucidate the significance of these associations, functional studies will be needed.

### MCTs and prostate cancer metabolism

At variance with other solid tumours [6,7,9], we did not find up-regulation of MCT1, MCT4 or CD147 in the plasma membrane of prostate cancer cells, proteins normally involved in the hyper glycolytic-acid resistant phenotype of cancer cells. On the other hand, there was upregulation of MCT2 and MCT4 in the cytoplasm of cancer cells, with a granular appearance. These findings suggest two hypothesis: either presence of alternative mechanisms that ensure acid efflux and maintenance of intracellular pH, or presence of an alternative metabolic pathway different from glycolysis that predominates in prostate cancer. Indeed, β-oxidation pathway is suggested to be up-regulated in prostate cancer [32] and α-methylacyl-CoA racemase (AMACR), an enzyme involved in branched chain fatty acid β-oxidation, is already being explored as a diagnostic marker of prostate cancer [33]. Additionally, MCT2, analysed for the first time in the present work, is involved in short-chain fatty acid transport and appears overexpressed and with strong staining in the cytoplasm of prostate tumour cells, with a granular appearance. These facts point to this MCT isoform as an important protein in prostate tumour cells, likely involved in some organelle function. In fact, the work of McClelland and collaborators provide evidence for a putative role of MCT2 in hepatocyte peroxisomal membrane [34].

## Conclusion

In the present study, we analysed the expression of MCT1, MCT2, MCT4, CD147 and gp70, in prostate cancer, corresponding adjacent non-neoplastic tissue, normal tissue and PIN lesions, and sought for associations with the clinico-pathological data of the patients. Our data provides novel evidence for the involvement of MCTs in prostate tumours. According to our results, we believe that MCT2 should be further explored as tumour marker and MCT4 and CD147 as markers of poor prognosis in prostate cancer.

## Competing interests

The authors declare that they have no competing interests.

## Authors' contributions

FB, JRV and CL were responsible for the study concept and design, study supervision, manuscript drafting and critical revision. NPG, VMG, CP, HP and JS performed the immunohistochemistry reactions and participated in the drafting of the manuscript. NPG, JRV, RMH were responsible for sample and clinic pathological collection and JRV and PM evaluated the immunohistochemical reactions.

All the authors read and approved the final manuscript.

## Pre-publication history

The pre-publication history for this paper can be accessed here:

http://www.biomedcentral.com/1471-2407/11/312/prepub
